# Examining Use Behavior of a Goal-Supporting mHealth App in Primary Care Among Patients With Multiple Chronic Conditions: Qualitative Descriptive Study

**DOI:** 10.2196/37684

**Published:** 2022-11-30

**Authors:** Farah Tahsin, Tujuanna Austin, Brian McKinstry, Stewart W Mercer, Mayura Loganathan, Kednapa Thavorn, Ross Upshur, Carolyn Steele Gray

**Affiliations:** 1 Institute of Health Policy, Management, and Evaluation Dalla Lana School of Public Health University of Toronto Toronto, ON Canada; 2 Centre for Medical Informatics Usher Institute University of Edinburgh Edinburgh United Kingdom; 3 Advanced Care Research Centre Usher Institute University of Edinburgh Edinburgh United Kingdom; 4 Mount Sinai Academic Family Health Team Mount Sinai Hospital Toronto, ON Canada; 5 Clinical Epidemiology Program Ottawa Hospital Research Institute Ottawa, ON Canada; 6 School of Epidemiology and Public Health University of Ottawa Ottawa, ON Canada; 7 Faculty of Pharmacy Chiang Mai University Chiang Mai Thailand; 8 Bridgepoint Collaboratory for Research and Innovation Lunenfeld-Tanenebaum Research Institute Sinai Health Toronto, ON Canada

**Keywords:** mobile health, mHealth, multimorbidity, chronic disease management, goal-oriented care, multimorbid, app, primary care, telemedicine, use, usability, human factors, behavior, sociobehavioral, health technology, mobile phone

## Abstract

**Background:**

Although mobile health (mHealth) apps are increasingly being used to support patients with multiple chronic conditions (multimorbidity), most mHealth apps experience low interaction and eventual abandonment. To tackle this engagement issue, when developing an mHealth program, it is important to understand the social-behavioral factors that affect patients’ use behavior.

**Objective:**

The aim of this study was to explore the social and behavioral factors contributing to patients’ use behavior of an mHealth app called the electronic Patient-Reported Outcome (ePRO). The ePRO app supports goal-oriented care delivery in interdisciplinary primary care models.

**Methods:**

A descriptive qualitative study was used to analyze interview data collected for a larger mixed methods pragmatic trial. The original 15-month trial was conducted in 6 primary care teams across Ontario, Canada, between 2018 and 2019. The eligibility criteria for patients were being aged ≥60 years with ≥10 visits within the previous 12 months of study enrollment. For this analysis, patients were classified as long-term or short-term users based on their length of use of the ePRO app during the trial. The Social Cognitive Theory by Bandura was used to categorize social-behavioral factors that contributed to patients’ decision to continue or discontinue using the app.

**Results:**

The patient-provider relationship emerged as a key factor that shaped patients’ experiences with the app and subsequent decision to continue using the app. Other factors that contributed to patients’ decision to continue using the app were personal and social circumstances, perceived usefulness, patients’ previous experience with goal-related behaviors, and confidence in one’s capability. There was an overlap of experience between long- and short-term app users but, in general, long-term users perceived the app to be more useful and their goals to be more meaningful than short-term app users. This observation was complicated by the fact that patient health-related goals were dynamic and changed over time.

**Conclusions:**

Complex patients’ use behavior of a goal-supporting mHealth app is shaped by an array of sociobehavioral factors that can evolve. To tackle this dynamism, there should be an emphasis on creating adaptable health technologies that are easily customizable by patients and able to respond to their changing contexts and needs.

**Trial Registration:**

ClinicalTrials.gov NCT02917954; https://clinicaltrials.gov/ct2/show/NCT02917954

## Introduction

### Background

Mobile health (mHealth) apps are being increasingly used to deliver care and support patients with chronic conditions [1-3]. Managing chronic conditions effectively is an ongoing task that often requires sustained support from an interdisciplinary team of health care providers. Continued involvement of multiple health care providers in supporting chronic disease management can be costly to the health system [4] and demands the time and resources of providers as well as their patients [5]. This management may be particularly challenging for patients with complex needs. Individuals with complex care needs are those who live with multiple chronic conditions (multimorbidity) [6] and experience additional health- and biopsychosocial-related challenges because of increased treatment requirements, reduced functional ability, and socioeconomic challenges [7]. To improve patient experience and tackle the issue of high health care burden and costs, mHealth is considered to be an effective and efficient solution [8]. mHealth offers an array of functionalities, which can include remote monitoring of patients’ vital signs and symptoms; ongoing and timely communication with multiple providers; and information sharing such as scheduled appointments, drug prescriptions, and renewals [9].

The positive benefits of using apps and web-based platforms to support complex patients is documented in the current literature [10,11]. For example, when patients with chronic illnesses use mHealth apps, they are more likely to engage in health-promoting behaviors such as fruit consumption and physical activity [12]. However, the benefits are more likely to be realized if the technologies are used as intended. Instead, most mHealth apps experience high attrition [13,14], defined as when an individual disengages from a technology-based intervention after initially committing to using the technology [15].

### Objectives

Attrition has been considered a major challenge in mHealth-based interventions [15-17]. Previous research has identified that only a small number of participants use mHealth apps in the long term, and most patients abandon the app after a short period [16,18]. The reasons behind high attrition vary. Critical factors that drive attrition can include available social support and capital, trust in technology, intention, and ability to use the app [19-21]. A meta-analysis evaluating the rates of attrition in mHealth interventions showed that many attrition-focused studies had relatively short intervention (<2 months) or follow-up periods [15]. This synthesis work suggests a need to advance knowledge of attrition by exploring sociocognitive factors that contribute to patients’ app use in the long-term and real-world settings [15]. To address this gap in the literature, this study explores community-dwelling patients’ perception of the long-term use of an mHealth app by applying the Social Cognitive Theory (SCT) by Bandura [22] to unpack sociocognitive factors that play a role. The research question informing this study is as follows: *What are the social and behavioral factors that contribute to continued or discontinued use of a goal-management app tailored for patients with complex chronic conditions?*

## Methods

### Description of the Electronic Patient-Reported Outcome Intervention

The electronic Patient-Reported Outcome (ePRO) tool is both an mHealth app and a portal that enables goal-oriented care delivery by facilitating goal creation and monitoring by patients with complex chronic conditions working in collaboration with an interdisciplinary primary care team [9,23,24]. User-centered co-design methods were used to develop the app through multiple iterations [25]. The co-design method was operationalized using inputs from patients with complex care needs, caregivers, and the primary care team [25,26]. The usability and feasibility of the app were assessed during usability testing [25] and exploratory trials [26] of ePRO. The findings from the exploratory trial informed the modification of the ePRO app to meet patients’ needs.

In a usability study of the ePRO trial, it was found that the app experienced gradual attrition of participants despite the tool scoring moderate usability [27]. The qualitative analysis presented in this paper was conducted to deeply explore social-behavioral factors that may be influencing patients’ low engagement with the ePRO app that were found in the usability study. Of note, ePRO is not an open-source app and was only available to study participants for the duration of the trial. A screenshot of the app interface can be found in a previous publication [23].

### Description of the Study Design

We conducted a descriptive qualitative substudy drawing on patients’ interview data collected as part of a larger 15-month multisite pragmatic stepped-wedge trial of the ePRO tool [23,24]. The trial registration number for the study is NCT02917954. Following the stepped-wedge trial design, 6 sites were randomized into 2 intervention clusters, and 2 different clusters received the ePRO intervention at 2 different time points. As a result, the first group used the ePRO app for 12 months after a 3-month control period, and the second group used the app for 9 months after a 6-month control period [23]. The qualitative descriptive approach seeks to present data as close to how the participant would understand the phenomenon as possible, referred to as “*staying close to the data.”* [28] This approach allowed us to present the patient’s direct description of their experience of the ePRO intervention and the factors that they perceive as contributing to their discontinuation or continuation of use without too many interpretive interferences from researchers [28]. Therefore, the findings of this study closely represent patients’ experiences with the intervention.

A 2-stage sampling strategy was used to recruit participants for the study. First, we recruited family health teams (FHTs), and then we recruited patients within each FHT. FHTs are designed to provide integrated, multidisciplinary primary care and are typically led by physicians or nurse practitioners [29]. A purposeful sampling strategy [30] was used to recruit 6 FHTs across geographically diverse areas (urban, rural, and suburban) of Ontario, Canada, from 2018 to 2019; this FHT recruitment process is described in detail in another publication [23,30]. The categorization of sites into rural, urban, and suburban settings was consistent with Statistics Canada’s definition of rurality [31]. The geographic location of the FHT was important for capturing the variation in the study participants. The eligibility criteria for FHTs were being an Ontario-based FHT and willingness to participate in the ePRO study. Ontario is the largest province in Canada, with the highest population density, and most services provided by primary care teams are funded by the Ministry of Health.

Quantitative data (surveys and chart audits) were collected from all 6 sites, whereas qualitative data were collected from 3 case sites [23,24]. At first, 67% (4/6) of the sites agreed to participate as case sites. However, 17% (1/6) of the sites dropped out because of low patient recruitment [23,24]. Patient interviews, demographic surveys, and research memos collected in these 3 case sites were used to answer the research question of this study.

### Participants and Interviews

The eligibility criteria for the recruited patients within the FHTs were being aged ≥60 years with ≥10 visits to the FHT within the previous 12 months. A total of ≥10 visits [32] and an age of ≥60 years [26] were chosen as both factors are considered as an indicator of complexity of this study population and were used as a recruitment strategy for the exploratory trial [26].

Using FHT electronic medical records, eligible patients were identified. The list of eligible patients was then given to FHT providers to assess whether the patients met the following additional criteria: (1) perceived willingness to engage in a conversation about goals of care, (2) ability to use a smartphone or tablet in English or having a caregiver who could do this on their behalf, (3) capability to provide consent to participate, and (4) willingness to complete surveys every 3 months thereafter until the trial concluded. Eligible patients were approached by their FHT staff (ie, care coordinators and administrators) and asked if they would be willing to speak to a research team member about the project. Recruitment occurred during a scheduled office visit or by phone. A detailed description of the recruitment procedure has been provided elsewhere [23,24].

Patients’ demographic information was collected through a survey at the beginning of the study. The first set of interviews was conducted at the midpoint of the trial, 4 to 6 months after the patients started using the app (the timing of the interviews depended on whether they were in the 12- or 9-month use group). The second round of interviews was conducted at the end of the trial. The purpose of the 2 sets of semistructured interviews was to explore patients’ overall experience with the ePRO intervention and how that experience changed over time. The semistructured interview guide addressed the following topics: (1) perception and experience of using the ePRO app, (2) patients’ relationship with their care team, (3) perception and experience of setting goals through ePRO, and (4) impact of ePRO on patients’ daily lives. Following the first set of interviews, the semistructured interview guide of the study was modified for the second set of interviews. Findings from the first set of interviews guided the iteration process for the semistructured interview guide and were decided by the research team members (FT [research coordinator], TA [graduate research assistant], JS [research coordinator], and CSG [research scientist and principal investigator with extensive qualitative research experience]).

The interviews were 25 to 40 minutes long and were conducted by 1 of 4 research team members (FT, TA, JS, and CSG). Each interview was audiotaped and transcribed using a commercial transcription service. Transcripts were checked for accuracy against recordings by a member of the research team.

### Ethics Approval

Ethics approval was received from the University of Toronto Health Sciences Research Ethics Board (33944) and the research ethics boards of the 3 participating primary care practices. All patient participants provided informed verbal and written consent before initiation of study activities.

### The Theoretical Framework for Data Analysis

Multiple theories and frameworks have been used to explore the relationship between patients’ social-behavioral factors and mHealth or eHealth use [13,32]. One such theory is the SCT by Bandura [33], which explains human behaviors through a model of interactions among behavioral, environmental, and social factors. This model has been used extensively to uncover which social and behavioral constructs may influence patients’ use behavior of an mHealth app [33-35]. Textbox 1 shows the 5 key domains of the SCT. SCT is particularly well suited to examine patients’ use behavior of an mHealth app such as ePRO as this app enables users to evaluate and monitor their goals over time and modify their behavior [23]. SCT also allowed us to understand the social-cognitive–related factors that contribute to the process by which patients decide to continue or discontinue app use. For example, one of the SCT domains, reciprocal determinism, is helpful to identify how personal, environmental, and behavioral factors can influence one’s decision to continue or discontinue app use. Similarly, the behavior capability and goal efficacy domains were helpful to identify how one’s skills and confidence can influence their decision on app use.

**Domains of Social Cognitive Theory (SCT) [22].**

**Reciprocal determinism**

- This constitutes the dynamic and reciprocal interaction of person (individual with a set of learned experiences), environment (external social context, technology, and aids), and behavior (responses to stimuli to achieve goals). In SCT, these components—behavior, environment, and individual—are seen as acting bidirectionally.

**Behavior capability**

- This constitutes a person’s actual ability to perform a behavior through essential knowledge and skills.

**Goal efficacy**

- This constitutes the level of a person’s confidence in their ability to successfully perform a behavior.

**Use reinforcement**

- The internal and external responses to a person’s behavior affect the likelihood of continuing or discontinuing the behavior.

**Outcome expectancies**

- This constitutes the anticipated consequences of a person’s behavior. Outcome expectancies can be health-related or not health-related.

This theory was used to guide data analysis to explore how complex patients’ personal beliefs and attitudes and physical and social environmental factors affected their engagement pattern (long-term and short-term app use) with ePRO. Although SCT can be used as an explanatory framework, it was applied in this study to help categorize factors influencing use and relate those to engagement patterns. During the interview debrief sessions, memoing activities, and initial reading of the transcripts, the authors (TA, FT, and CSG) agreed that SCT demonstrated a fit with the interview data. As we chose SCT as the right analytical tool based on emerging interview data, we did not encounter the challenge of forcing data into categories.

A combination of 2 techniques was used to analyze the study data. In stage 1, the transcripts were inductively coded by 2 analysts (FT and TA). During the analysis, the research team met to discuss the identified codes and resolve any coding discrepancies. After coding 4 transcripts, the team decided that the coding scheme was appropriate. We reached data saturation after coding 12 transcripts. Data saturation was determined when no new codes emerged from the transcripts [36]. After coding all 22 transcripts, the codes were mapped onto the SCT categories, meaning that inductively identified codes were plotted within the SCT categories to form themes [37].

The first stage allowed us to see the social and behavioral factors related to use. However, to see how these factors related to each other and changed over time, we engaged in the second analysis stage of restorying.

Restorying is defined as the method of rewriting participants’ oral data temporally to draw a link between previous experience and subsequent experiences [38]. Restorying revealed how themes related to each other and changed over time. It also allowed us to more clearly see pattern differences across different user groups (short- vs long-term users), which allowed us to more directly address our question regarding social and behavioral factors that were related to continued or discontinued use. Restorying allowed us to generate exemplary narratives of long- and short-term app users as a means to illustrate these patterns. The definitions of long-term and short-term app users are described in the following section.

To restory patient data, 2 analysts (FT and TA) constructed a matrix of themes that distinguished between long-term and short-term app users (Table S1 in Multimedia Appendix 1 [38-40]). After examining both columns of long- and short-term app users, 2 research team members created a storyline for each group that captured the experience of the overall group. The restorying allowed us to see the connections between SCT constructs within the context of patient use of ePRO and how those connections influenced use progression over 15 months [38]. Although one of the major criticisms of SCT is that it does not recognize the wider social structure that influences an individual’s use behavior [39], the analytic method of restorying addresses this challenge by highlighting the social contexts influencing use behavior over time. A detailed description of the 2-stage method can be found in Multimedia Appendix 1.

To enhance the rigor of this study, the researchers undertook several strategies to increase the credibility and trustworthiness of the findings [41]. The research team members met regularly to discuss codes and findings. In addition, throughout the restorying process, both researchers discussed the accuracy of the storyline. Member checking [36] was conducted with study participants to examine the accuracy of the 2 storylines and the overall interpretation of the study findings. Furthermore, having 2 data analysts helped ensure the dependability of the findings [40]. Both analysts (FT and TA) had graduate-level training in qualitative data analysis. In addition, one team member (CSG) provided supervisory support during the analysis.

### Categorizing Patients Into Long-term and Short-term App Users

On the basis of patients’ app-automated use logs, patients were classified into 2 categories: long-term users and short-term users. Of the 22 interviewed individuals, 9 (41%) were short-term users and 13 (59%) were long-term users. Participants who did not use the ePRO app after initial onboarding or used it for <3 months were categorized into the “short-term user” group. By contrast, the participants who used the ePRO app for >3 months were categorized into the “long-term user” group. The 3-month cutoff period was determined because the app experienced a sharp decline in use at 3 months [27]. This 3-month cutoff period is also consistent with the previous literature [41].

## Results

### Overview

There were 44 study participants in the larger pragmatic trial, with 37 (84%) from the 3 case sites. Of the 37 patients who were invited to participate in the interviews, in total, 22 (59%) were interviewed. Of the 22 interviewed patients, 17 (77%) participated in both interviews, 3 (14%) participated in only the midpoint interviews, and 2 (9%) participated in the last interview only. A total of 41% (15/37) of the participants did not take part in the interviews because of scheduling issues, illness, being out of the country when the interview was scheduled, or not responding to interview requests.

### Demographic Description of the Participants

The demographic information of the study participants can be found in Table 1. The mean age of the 22 interviewed participants was 75.1 (SD 5.67) years, and 45% (10/22) self-identified as female. We also reported the demographic characteristics of the participants who did not take part in the interviews (15/37, 41%) to show any demographic differences between the interviewed and noninterviewed groups. It is worth noting that there were more noninterviewed participants in the lowest income quintile. However, we did not identify any statistically significant demographic differences between the interviewed and noninterviewed participants. We conducted descriptive statistical analyses (2-tailed Student *t* test for continuous variables and Mann-Whitney *U* test for categorical variables) to explore the differences between the groups (interviewed and noninterviewed and short-term and long-term).

**Table 1:** Demographic characteristics (N=37).

Variable		Interviewed participants (n=22)	Noninterviewed participants (n=15)
Age (years), mean (SD)		75.1 (5.6)	71.14 (6.5)
Sex—female, n (%)		10 (45)	5 (33)
Smartphone comfort level score, mean (SD)^a^		2.17 (1.4)	3.64 (1.4)
Number of chronic conditions, mean (SD)		4.88 (2.1)	3.07 (1.8)
**Family income, n (%)**			
	CAD $0 to $29,000 (US $0-$21,310.40)	1 (5)	4 (27)
	CAD $30,000 to $59,000 (US $22,045.30-$43,355.70)	7 (32)	4 (27)
	CAD $60,000 to $89,000 (US $44,090.60-$65,401)	3 (14)	3 (20)
	>CAD $90,000 (US $66,135.90)	4 (18)	3 (20)
**Education, n (%)**			
	Lower than high school	2 (9)	2 (13)
	High school	2 (9)	4 (27)
	Some college or university	4 (18)	3 (20)
	University (undergraduate or graduate)	4 (18)	5 (33)

^a^The range of the smartphone comfort level score is 1 to 5. A higher score indicates a higher comfort level with a smartphone.

### Summary Description of the Themes

The patient interviews revealed insights into the factors that influenced patients’ decision to continue or discontinue app use. When discussing their use of the ePRO app, patients identified what encouraged them to use the app, including factors relating to their social and clinical relationships, capability to use the app and perform goal-related activities, and their expected outcomes from the ePRO app. Table 2 summarizes these factors in relation to the SCT domains. In addition, to provide a contextual understanding of these factors, long-term and short-term user narratives generated by restorying the data are first presented (Textbox 2), followed by a more in-depth exploration of each factor as it emerged in the full data set.

**Table 2:** Description of the themes.

Category and subcategory		Exemplary quotes	
		Long-term user	Short-term user
Reciprocal determinism^a^		“They [care team] always know what to do with me, so there was no problem setting goals because they know that I am trying to be active and healthy. and I kept using it (ePRO app) daily because I know they (care team) are watching my data.” [Female, patient 18]	“I just did not know if anyone is looking at my data, there was no communication from you guys [research team] or my nurse or doctor here. There was no feedback for me about my data, so I felt like I am talking to the void when I was putting my information in. I would like to know if I was doing well or not. It would be helpful to talk to others (peers) about our goals, to see who else is doing the same thing as me and how they are feeling.” [Female, patient 16]
**Goal efficacy, behavior capability, and outcome expectancies^b^**			
	Subtheme 2a—confidence and skills in goals	“When my dietician first asked what goal I wanted to set, I knew it would be tracking my everyday walk, I knew it would be easy to keep up at because I have been doing this for long time. But ePRO made me more accountable, I wanted that accountability. I liked how the device asked me if I have achieved my goal for that day. Clicking yes to that felt good and I kept doing that.” [Male, patient 7]	“Setting any goal was hard for me because my conditions flare up here and there and throws me off my routine. So I wasn’t sure how well I can keep up with the goals...I sprained my ankle in last winter so then I was off my walking for 5 weeks. Considering all these troubles, I didn’t work on my goals, and the app became redundant because what would I track. When the app asked Did I achieve my goal for the day, I did not want to keep saying no.” [Male, patient 2]
	Subtheme 2b—confidence and skills in technology	“I expected the app to have some direction for me about how I was doing on my goals, it was nice to see what I was accomplishing weekly basis. No complaints about the app, very easy to use...nothing complicated that anyone will have difficulty with...But I have used computer all my life for work so using this phone or any other phone is not a problem.” [Female, patient 3]	“The small fonts or buttons in this phone [ePRO] was trouble...but I thought I will get used to it (the phone) but did not at the end. I was sometimes working on my goals but could not record it on the phone, so I lost interest in the phone...then I forgot about my goals too because I was not tracking it or doing anything about it.” [Male, patient 21]
	Subtheme 2c—outcome expectancies	“The main reason I enrolled-I wanted to stay on track of my goals and feel healthier over time-I thought the app was helpful to keep me on track.” [Male, patient 1]	“When my doctor suggested this app, I did not know what to expect because there is nothing important, I need to work on, in my opinion anyway. My doctor suggested some goals but nothing very important...I could not make a purpose of it (ePRO).” [Male, patient 11]
Use reinforcement^c^		“I was bedridden so [provider’s name] she was ‘gung-ho’ that I join her walking group for my recovery. And she said, “why don’t you try this new thing we are doing, this will be good for you?”. And She was right, it was nice to have the app because I know every Monday, I will have to say how many times I walked last week, so I tried to go out over weekends...She was there for me throughout, walking alongside me in every walking group.” [Female, patient 6]	“My doctor did not think ePRO was helping me that much, because both of us thought I am doing fine without it, everything [diabetic symptoms] was on track, so we decided maybe I do not need it.” [Male, patient 17]

^a^This domain refers to the dynamic relationship between individual, context, and behavior.

^b^This domain refers to individuals’ confidence and skills in achieving their goals in the electronic Patient-Reported Outcome app and the perceived usefulness of the app.

^c^This domain refers to the internal or external responses that encourage or discourage behavior change.

**Long-term and short-term user stories.**

**Elaine: a long-term user story**

Elaine considers herself to be a healthy individual whose diabetes symptoms are well managed through diet and exercise. She thinks of herself as “lucky” to have great health care providers who have helped her manage her symptoms for the past 2 and a half years. She has multiple other chronic conditions such as chronic pain and hypertension, but controlling diabetes symptoms is her foremost priority as she heard it can affect her other conditions. At first, she joined the electronic Patient-Reported Outcome (ePRO) study because her dietician at the family health team encouraged her to do so (*Social Cognitive Theory [SCT] domain: reciprocal determinism [social support]*). After talking to her dietician and talking to the ePRO study recruiter, Elaine agreed that ePRO would be a good addition to be more accountable toward her health-related goals (*SCT domain: outcome expectancies*). With her dietician, she decided on 3 goals that she always thought would be important to lead a healthy lifestyle. Elaine’s goals were (1) lowering daily sugar intake, (2) joining walking programs with her peers facilitated by her dietitian, and (3) swimming every weekend in the local community center. She felt confident that she would be successful in achieving these goals as she had always been self-disciplined (“No TV from 9 AM to 6 PM”) and kept a personal calendar to track her physical activity level. She also considers herself not in frail health, so she did not think that working toward those exercise-related goals would be hard for her (*SCT domain: goal efficacy and outcome expectancies*). She had also been working on those goals before the ePRO intervention, so she was confident that she had the necessary skills to work toward her goal (*SCT domain: behavior capability*) and thought ePRO would be beneficial for her to track those goals (*SCT domain: outcome expectancies*).

Elaine considers herself technologically savvy. However, she experienced a few technological difficulties while using ePRO. The most challenging one was being logged out of ePRO after taking a break from the tool during Christmas time when she visited her family in Scotland for 15 days. After not using ePRO while she was away, Elaine was locked out of the app. After returning from her holidays, she contacted her dietician to resolve the issue (*SCT domain: reciprocal determinism*). Her dietician asked her to contact the research team as she could not fix the technical issue for Elaine. Elaine’s technical issue was resolved in 2 days, and she continued to use the app until the end of the study. In the final reflection, Elaine believed the app was good for her to be accountable toward her goal, and she derived satisfaction from that accountability. At the end of the study, Elaine planned to continue to track her goals through her calendar, which was how she tracked her goals before using the ePRO app. She thinks ePRO would benefit from having a communication feature. That way, she could communicate with her peers who are also using ePRO and working toward similar goals.

**Josh: a short-term user story**

Josh is a man aged 76 years with several concurrent chronic conditions, including diabetes, hypertension, and arthritis. Josh considers himself to have a fair understanding of his ailments and considers that his conditions are fairly well managed. Josh is the primary caregiver to his wife, who is ill. As a result of this caregiving role, Josh finds that he does not often have time to participate in social groups such as walking groups offered through his local community center (*SCT domain: reciprocal determinism [social support]*). Josh is a patient at a family health team where he has access to both primary care and allied health services. At the suggestion of his family physician, Josh agreed to participate in the ePRO study (*SCT domain: reciprocal determinism*). However, he did not expect the app to be useful as he considered himself to be “tech illiterate,” so he did not think he would be able to use the app without his wife’s help, and he did not think he had any important goals to work toward as he already had a healthy lifestyle (*SCT domain: behavior capability and outcome expectancies*).

In addition, Josh was hesitant to set a goal as he had never had a health-related goal before and was uncertain about whether he had the necessary skills or discipline to keep up with a specific goal (*SCT domain: behavior capability*), so he was not sure if ePRO would add value to his life (*SCT domain: outcome expectancies*). However, with assistance from the ePRO study team and his family physician, Josh created the following SMART goals: (1) eat at least one fruit every day and (2) walk for at least 10 minutes every day.

At the beginning of the study, Josh completed his check-in questions regularly. Over time, Josh began checking in on the app less and less, eventually not using the technology at all. When the ePRO study team reached out to Josh, he stated that he forgot his password and was unable to log in to the ePRO platform, so he did not use it. Although Josh describes himself as “computer illiterate,” he found the ePRO app and web platform easy to use. Josh also found that, whenever he met with his health care provider, they did not discuss his goals but rather spoke about his medications and management of his conditions, resulting in goal setting becoming less of a priority (*SCT domain: behavior enforcement*). Josh found ePRO to be good for self-monitoring, but he did not find the technology useful for communicating with his health care team.

A major decision that was made during the analysis was to collapse 3 SCT domains—goal efficacy, behavior capability, and outcome expectancies—into one as it was identified that patients’ confidence in their goal and technological skills was linked to the anticipated outcome of the ePRO app. Previous studies on goal-setting behavior have also identified that, in a real-world setting, individuals’ confidence in health-related goals is confounded by their outcome expectancies, capability and skill level for carrying out various goals and activities, and technological and health literacy [39,42]. Applications of SCT in the literature have found that the relationship between multiple domains of SCT is multidirectional rather than unidirectional, as suggested by the original SCT, meaning that SCT domains can be both antecedents and consequences of each other [39]. For example, individuals who receive no feedback on their performance may lose motivation to continue engaging in a task and anticipate negative outcomes from their performance. Hence, in this analysis, we grouped these 3 domains together to retain the interrelationship as factors that contributed to patients’ use behavior: confidence and skills in goals, confidence and skills in technology, and outcome expectancies.

### Description of the Themes

#### Theme Overview

In this section, we elaborate on the themes identified in the data according to the SCT domains. Some domains had richer information than others. For example, the themes related to reciprocal determinism, goal efficacy, and outcome expectancies had more nuanced data compared with the other 2 themes, which were behavior capability and use encouragement.

#### Theme 1: Reciprocal Determinism

Reciprocal determinism focuses on the dynamic interaction between person-context behavior and the influence of this dynamic interaction on individuals’ behavior. As demonstrated in the long- and short-term user narratives as well as in Table 2, social and clinical relationships are key factors for the continued use of the ePRO app.

In total, 46% (6/13) of long-term users described their longstanding relationship with their primary care providers as being beneficial to setting meaningful goals:

I got lucked out with my providers, they will always know exactly how to deal with me and keep me out of the hospital, which is my main goal. My doctor knows that my nurse and dietitian here (primary care team) know that, so it was easy to set those goals to keep my blood sugar low.

Long-term user, male, patient 7

Short-term users also described a good relationship with their providers. However, 44% (4/9) of short-term users described that their providers did not discuss the ePRO app during their clinic visits. Participants listed the following reasons for not discussing the ePRO app with their providers: clinicians’ heavy workload, not having enough time during the visit, feeling that it was unnatural to discuss the app during a regular clinic visit, and feeling that their goals were personal work and did not fall under providers’ responsibility. A participant described the following:

Dr. [physician’s name] is great, but he is really busy, so I did not want to waste his time talking about my walking schedule. He needs to check my blood pressure level; I would not bring up how many times I walked last month. Feels irrelevant for him to know that.

Short-term user, male, patient 2

Another way the patient-provider relationship influenced app use was when patients faced any sort of technical error in using the app or had to modify their goals after the initial goal-setting process. Specifically, long-term users were more likely to reach out for support and tended to report more instances of connection with their providers regarding the ePRO app. Some of the common technical challenges were (1) being logged out of the app because of prolonged inactivity, (2) forgetting passwords, and (3) inability to modify goals based on patients’ needs. In terms of modifying goals, ePRO did not allow patients to modify their own goals, so primary care providers had to modify the goals for them. Therefore, when patients needed to modify their goals, they were uncertain about how to do that:

After they (government) changed the number of blood glucose tests I can do per week, my goal had to be changed because I wanted to test my glucose level daily but after they changed it, now I only test twice a week, but I still it report it on the phone just not daily. And my nurse over here changed it (frequency of reporting) for me.

Long-term user, male, patient 12

When faced with these technical difficulties or needed modifications, patients either abandoned the ePRO app or reached out to their health care providers or research team to solve the issue. Most long-term users (7/13, 54%) chose the latter option:

I was locked out of the app when I was on vacation...after I got back, I contacted the dietician over here (care team), and she connected me to you guys. Everything got resolved within 2 days, I kept using it.

Long-term user, male, patient 1

Short-term users, by contrast, decided to abandon the app and did not reach out for support when they faced similar technical difficulties:

It would be good if I could change my goals in the app because walking 5 km is what I set out to do at the beginning. It was too ambitious of a goal in this bad winter. I never reached 5 km, so I never had anything to report on the app...I did not reach out to my nurse practitioner, I guess I forgot about it (ePRO) for a while, and then I asked you (research team) to take it away.

Short-term user, female, patient 22

Both long- and short-term users also reflected on the fact that their relationships with peers and their communities could influence their app use behavior. For example, a patient discussed that being able to communicate with their peers would be useful in understanding others’ experiences with the ePRO app:

Sometimes I felt that the app does not give me enough feedback. There could be more photos, a thumbs up if I did well. I’m a unique person so when I found I felt that way I thought, well I wonder if anyone else is feeling that way. So, communicating with other people that are using it without divulging your specific things would be nice.

Long-term user, female, patient 19

Importantly, unexpected changes in these relational contexts also influenced patients’ use behavior, for example, a sudden transition to a caregiving role, a move away from social ties, or a divorce:

After my marriage fell apart, I moved to this area with my partner and I have to keep going back to the city to meet my friends, which makes it harder for me to meet people here. I am currently in an anxiety support group here, but I went off track with my other goals. I check the app (ePRO) sometimes but not regularly because I have nothing to report on.

Long-term user, female, patient 14

#### Theme 2: Goal Efficacy, Behavior Capability, and Outcome Expectancies

##### Overview

Patients’ confidence, skills, and anticipated outcomes from the app influenced their use behavior. Although presented as distinct domains in SCT, data from this study suggest that the domains of goal efficacy, behavior capability, and outcome expectancies are linked.

The restorying work reveals these connections, which are best represented in the long- and short-term user narratives in Textbox 2. However, some participants’ accounts also show that individuals’ confidence in themselves to achieve goals (perceived goal efficacy), skills necessary to use the app (behavior capability), and commitment to engage with the app to achieve set goals (outcome expectancies) are intertwined and influence each other. These outcome expectancies were also related to app functionality. This collapsed theme consisted of the following subthemes: (1) patients’ confidence and skills with goals and their impact on ePRO use (subtheme 2a—confidence and skills with goals), (2) patients’ confidence and skills in using technologies and their impact on ePRO use (subtheme 2b—confidence and skills in technology), and (3) patients’ expected outcome from the ePRO app and its impact on their use behavior (subtheme 2c—outcome expectancies).

##### Subtheme 2a: Confidence and Skills With Goals

This subtheme demonstrates patients’ descriptions of how their confidence in their goals and their skills to achieve the goals influenced their ePRO use behavior. Previous goal-setting experience and familiarity with goal-related tasks influenced patients’ confidence in achieving the goals set in the ePRO app. Patients who had been working on a goal for a long time were more confident in their skills to achieve a goal. A total of 38% (5/13) of long-term users had already been working on a number of health-related goals before enrolling in the study and had been tracking their progress using electronic or paper-based tools such as calendars, wearable technologies, and handwritten notes. For these participants, the ePRO app was an additional electronic way to track their goals. These participants demonstrated confidence that they had the necessary skills to set appropriate goals and achieve them with the use of ePRO and, because they had the confidence and skills, they also had better outcome expectancy from the ePRO app:

I did pretty well in terms of crushing all my goals...because I already had the same goals, I was already continuing with the exercise program. So, it (ePRO goals) was just a continuation. I just kept up with the same tasks, swimming, walking that I was doing before joining your study.

Long-term user, female, patient 3

By contrast, patients who did not have any previous goal-setting experience reflected on the fact that setting a meaningful goal was difficult for them. Consequently, their providers had to suggest some goals for them, but some patients found that those goals were not personally meaningful. In these cases, not having previous goal-setting experience negatively affected patients’ ability to set meaningful goals, which in turn affected their use behavior:

I’ve never had health goals before, so could not come up with one when they (health provider) asked me what I want to put in here (ePRO app). I got some kidney conditions, so my doctor suggested I set daily goals of drinking eight glasses of water and tracking them. I did not think I need to track it; I remember it anyway. I don’t need a phone to tell me I need to hydrate. I did not think the goal was anything important for me to track on a phone.

Short-term user, male, patient 11

In terms of individuals’ confidence in achieving their goals, some long-term users (6/13, 46%) indicated that their traits, such as “will-power,” “self-discipline,” and “motivation,” boosted their confidence that they would be able to reach their goals:

It [achieving health goals] has nothing to do with the phone [ePRO app]. It has everything to do with the person. You have to be determined that you are going to walk. And you’re going to set your goal—you’re going to walk a block and you’re going to walk back. You have to have determination. You have to have the willpower to say, I’m going to do it and that’s it. ePRO is not going to do it for you, but it was good to have to see my progress. I thought it (ePRO) was a neat way to see how I am doing.

Long-term user, female, patient 6

In addition, patients reflected on the fact that their confidence and skills in achieving a goal changed over time depending on their health. When patients felt that they were not able to achieve their goals because of health and life circumstances and they did not have “enough” to report on the app, they discontinued using it:

Initially, I set up my goal to go 3 miles walking every day. But after my surgeries and my accident, there was no way I could do it. I was barely getting out to walk my dogs. I was falling short every day and it made no sense for me to use the app, I just felt sad that it [ePRO] kept showing me I was not the go-getter anymore. I did not know how to pause it [ePRO].

Short-term user, female, patient 15

##### Subtheme 2b: Confidence and Skills With Technology

Not surprisingly, patients who did not think that they had the necessary technological skills to use the ePRO app discontinued their use.

Several patients (14/22, 64%) discussed that they were technologically savvy enough to be able to use the app:

I found the app to be user-friendly, very clean, nothing too difficult, but I am good with computers and all that stuff, a tech-junkie. I use computers, phones, iPad all the time.

Long-term user, female, patient 19

Some participants (4/22, 18%) stated that they needed help using the ePRO app as often the fonts were too small:

I never had to use the computer for my work so never learned it. Now I got muscular dystrophy, so the fonts were way too small for me, so I did not use the app at all. I used the app [ePRO] on my computer, but I am not very good at it. My wife must help me a lot. I cannot even send an email; she will just do it for me. I ended up not using it [ePRO on the computer] at all.

Short-term user, male, patient 17

##### Subtheme 2c: Outcome Expectancies

Patients described their anticipated outcomes from the ePRO app. Typically, for long-term users, ePRO seemed like a beneficial addition to their health. A long-term user described that, while enrolling in the study, they anticipated that ePRO would make them more accountable toward their goals:

I wanted to get off my oxygen tank, I do not want to lug this machine everywhere. So I need to drop some pounds...by walking, exercising...I thought this phone would show me how I am doing, am I doing it too much, am I getting any good.

Long-term user, female, patient 20

By contrast, 33% (3/9) of the patients who were short-term users described that they discontinued using the app as they did not think that the app was “well-developed” to be implemented in the real world. Therefore, they did not think that the app would be a beneficial addition to their lives. A short-term user described their dissatisfaction with the functionality of the app:

I think that’s all [research on people taking control over their health] a great idea I just feel that the actual implementation isn’t as far advanced as it needs to be for it to work effectively, at least for me. I use my fitbit anyways to count my steps which is far better because that watch automatically counts my steps. I could not see any use for it [ePRO app] to work on my goals. I did not see any benefit for my health from it.

Short-term user, male, patient 10

#### Theme 3: Use Reinforcement

The use reinforcement domain of SCT suggests that internal and external factors such as internal satisfaction or external rewards can encourage or discourage individuals’ behavior change. In total, 38% (5/13) of long-term users reported that they felt a sense of accomplishment (ie, internal reward) when they were able to “check off” their goals in the ePRO app. The app had the following question—“did you achieve your goal yesterday?”—and patients had the option of reporting yes or no. Some patients (6/13, 46%) found this exercise rewarding:

Well, to be honest, the only thing it did was—I do it [check off the list], used to do it every Monday morning, and it focused me on not smoking. That was the motivation every Monday morning, you know.

Long-term user, female, patient 20

Some short-term users (2/9, 22%) identified that they had already used many other legacy devices such as calendars, notebooks, cell phones, and glucose monitoring devices. These participants found reporting the same measures in 2 different tools to be redundant, and they did not think of the ePRO app as an important addition to their health-related goals:

I am an old school paper-pencil, calendar on refrigerator person, so that helps me to visualize my progress every day. I see them every day before breakfast, so I know what I had to do that day. The phone [ePRO] just stayed on my night table.

Short-term user, female, patient 22

An unexpected external influence can be discouragement from providers. Among the 9 short-term users, 22% (2/9) of the participants reported receiving advice from their providers to discontinue the use of ePRO. The factors that contributed to providers’ discouragement were patients’ frail health, patients’ anxiety with the app regarding not being able to reach their goals, and changed health-related priorities:

My breathing issue has gotten worse in winter so I was not working on my goals anymore...When I told her [health provider] that I am worried about not reaching my goal, I feel anxious that I am not reaching my goal, she said “just forget about it [ePRO] for now, let’s get back you to feeling good first,” so I thought okay one thing off my list. I felt better.

Short-term user, female, patient 13

### Long-term and Short-term User Stories

The 2 narratives presented in Textbox 2 offer a composite understanding of long- and short-term users of the ePRO app, linking elements of the stories shared by different participants to SCT domains.

## Discussion

### Principal Findings

This study used descriptive qualitative methods and restorying analytic techniques to explore the social and behavioral factors contributing to patients’ use behavior of the ePRO tool. Study findings show that patient-provider relationships, patients’ social relationships, and patients’ personal circumstances play a central role in their decision to continue or discontinue the use of the ePRO app.

Leveraging SCT as a tool for data analysis, we were able to identify social-behavioral factors that contribute to patients’ decision to continue or discontinue app use, such as their social and environmental factors and relationships (domain 1); confidence and skills in using technology, confidence and skills in setting and achieving goals, and expected outcomes from the intervention (domain 2); and encouraging factors (domain 3). Study data reveal that the SCT constructs of goal efficacy and behavior capability are also importantly related as capability and skill influence perceived confidence in completing a task. This interrelationship makes sense theoretically. SCT suggests that performing a behavior successfully increases individuals’ confidence in their ability to accomplish goals as they believe that they have the skills to achieve goals through behavior change [22]. In addition, performing a behavior successfully also affects one’s outcome expectancies as one believes that they have the skills and confidence to receive benefits from an action [22,43].

The stories show the themes of the interactions and links between concepts that the descriptive analysis could not. For example, an important interpretive theme that emerges from Josh and Elaine’s stories is that patients’ confidence and previous experience in goal setting influenced their capability and expectations from this goal-oriented intervention. Josh and Elaine approached their goals with varying degrees of experience, confidence, and attachment. For example, Elaine’s previous experience with goal setting helped her feel more competent and skilled in achieving future goals, which subsequently increased her intention to track goals through ePRO, whereas Josh’s lack of experience with goal setting made it challenging for him to make meaning of his goal, which translated into his reduced interest in tracking goals through ePRO.

Furthermore, the stories also show, in an interpretive manner, an important divergence in how long- and short-term users react to technical errors. App-related technical errors are ubiquitous, and many app-based interventions experience significantly high attrition after users experience an error [44]. As such, it is important to explore patients’ strategies to mitigate risk and what factors contribute to their motivation to resolve such technical errors [24,45,46]. The patient-provider relationship emerged as an important mitigating factor when resolving technical errors. In Elaine’s story, her strong relationship with her providers, the meaningfulness of her goals, and the satisfaction obtained from achieving goals influenced her motivation to proactively troubleshoot the problem and return to the app. This was a common occurrence among many long-term users, who would more readily troubleshoot technical errors with their primary care providers. Although this study provides an initial indication of the influence of the patient-provider relationship on technology use behavior, future studies should be conducted to determine the strength of this influence [47]. By contrast, for Josh, the combination of technical error and lack of meaning of his goals contributed to discontinuing app use. This finding shows that participants’ goal-setting success was related to user experience with the app. If participants face difficulties using the app interface, they may abandon the goal-tracking exercise altogether, as demonstrated in Josh’s story. In summary, factors such as the patient-provider relationship and app user experience can play an important role in a patient’s decision to continue or discontinue using a goal-oriented app.

Another important study finding that emerged from the interview data is the importance of meaningful goal setting for an effective behavior change intervention. Hence, when setting patients’ goals, a strong focus on patients’ perception of the meaningfulness or fit of the goal in their daily lives should be accounted for as this meaningfulness of the goal can influence not only behavior change but also patients’ adherence to a newly adopted technology [48,49]. This goal-oriented conversation between the patient and provider should also include an exploration of goal-setting and goal-monitoring tools that the patient may already be using, such as calendars, health-monitoring devices, or personal phones, as the study data suggest that often patients prefer devices and tools that they are familiar with rather than adopting a new tool [50].

### Comparison of Themes With Previous Research

The findings of this study support previous study findings that health technologies are often discontinued and abandoned when they lack features of meaningful customization and is not part of users’ already existing devices such as personal phones [50]. In addition, the study findings suggest that health-related goals change over time for patients with multiple chronic conditions, so designing apps that offer patient-driven customization and modification techniques will be helpful in repurposing the same technology at multiple time points of the life cycle. For example, patient 15 shared that their ability to achieve their goals changed over time because of emerging health issues, but they were unsure of how to modify the goals in the ePRO app. This design feature in ePRO was intentional based on a previous exploratory trial of the app (which was <4 months) [26]. In the exploratory trial of ePRO, it was found that the patients preferred provider consultations while changing their goals; hence, the app required the providers to modify goals on behalf of the patients. However, in this longer pragmatic trial of ePRO in which patients used ePRO for 9 to 12 months, patients preferred to modify their goals on their own, as demonstrated in these study findings. This contradiction may be due to the prolonged use of ePRO; for example, with prolonged use, patients’ confidence in using the app changed, which in turn helped them feel like they could take charge of their goals. This finding demonstrates the importance of longitudinal evaluation of mHealth apps compared with a shorter follow-up time as patients’ confidence, skills, and health needs from the app change over time, which may not be captured in a shorter trial [15].

For example, previous studies with shorter follow-up periods have identified that factors such as health literacy, motivation, capabilities, social and environmental structures, and social support have an impact on mHealth engagement [51,52]. However, this study shows that patients’ motivation, capability, and social and environmental factors change over time. A systematic review of mHealth interventions for patients with depression supports the finding that patients’ engagement with interventions changes over time [53], perhaps because their treatment needs and goals change over time. These changing needs of patients from their mHealth app interventions and their impact on their use behavior is further supported by another study conducted among patients with chronic illnesses [18]. Thus, we need to consider how our technologies can adapt to how users evolve over time.

In the current chronic care paradigm, the task of goal management is often left to the patients [3,54]. Our study findings highlight that those discussions regarding goal-oriented care are a one-time occurrence for study participants, which was facilitated by introducing the ePRO app. After setting goals with patients, providers often leave it up to patients to be responsible for their own goals. By contrast, patients do not bring up the topic of goals in their discussion as they perceive that their providers “are too busy” to attend to patients’ goals and providers’ time could be better spent on other condition-related concerns. This study finding reflects that there is a need for an ongoing conversation between the patient and provider about patient-centered goals to ensure that the goals and associated devices and tools are appropriate for the patient’s needs and serve the purpose that the goal or device set out to do. Similarly, the interview data suggest that patients considered that their providers’ enthusiasm for the ePRO intervention was important and influenced their interest in two ways: (1) monitoring of patient data by providers, which was considered important, and (2) providers’ encouragement to keep using the ePRO app [55]. This finding highlights the need for further education and training tools for health care providers on how to effectively have a goal-oriented conversation with patients and within interprofessional teams [10,56].

### Strengths

The descriptive qualitative approach of this research allowed us to identify multiple social-behavioral factors that influenced patients’ enrollment in the study and subsequent discontinuation or continuation. In addition, by using a restorying method, the findings were interpretive, allowing for the identification of nuanced patterns and interrelationships between identified themes. Furthermore, the longitudinal timeline of the study (15 months) allowed us to explore the factors that contribute to patients’ use behavior in the long term, which is underexplored in the current literature [15]. Finally, as the SCT by Bandura [22] has been widely used to explore an individual’s behavior and actions toward health-enhancing behavior, we were able to compare the findings of this study with previous literature [22,33,43,57]. For example, previous studies have identified that patients’ self-efficacy, motivation, capacity, social and environmental influences, and perceived consequences affect their use behavior of an mHealth app.

### Limitations

Owing to scheduling conflicts or loss to follow-up of participants, we were not able to interview all of them at either time point. As a result, a potential limitation of the study is that those who participated in the interviews may be unique as compared with those who chose not to. However, the sample size was too small to assess whether the difference between the 2 groups was significant. However, the interviews that were conducted were in-depth and provided rich information. Furthermore, the patient population represented in this study was recruited from only 3 of the 6 FHTs involved in this study. It is possible that some additional findings may have been obtained by looking across all 6 sites. However, the sample in this study represented 59% (22/37) of the total participants in the larger study. As is the case with case study research, it is also possible that findings may not be transferable to other models of primary care such as community health centers or solo practice environments. Furthermore, the participant demography suggests that the study patient population was less complex and well resourced, meaning that, on average, patients had a low number of chronic conditions and high income and educational attainment levels, which might not be representative of general complex patients. Therefore, the findings of this study may not be transferable to patients living in resource-poor communities or who have lower income or education levels. In addition, the underrepresentation of low-income individuals is a common occurrence across multiple research studies and requires attention in study design to facilitate this population’s participation [58].

### Conclusions

In many cases, mHealth or any health innovation will have expected impacts if people use it as intended. To better predict, explain, and increase the actual use of innovations, we need to understand why different target user groups continue or discontinue the use of an innovation. This study identifies that multilevel factors contribute to complex patients’ decision to continue or discontinue using a goal-oriented app. In addition, our findings show that there is a need for ongoing, productive patient-provider interactions to set and modify patients’ goals according to their changing health and social needs. Future research should consider patients’ social and behavioral contexts when implementing mHealth apps and similar technological interventions for complex patients.

## Appendix

Multimedia Appendix 1 Additional information about data analysis.

Multimedia Appendix 2 CONSORT-EHEALTH checklist (V 1.6.1).

## Abbreviations

ePRO: electronic Patient-Reported Outcome
FHT: Family Health Team
mHealth: mobile health
SCT: Social Cognitive Theory
