# Supplementary material for: Examining Use Behavior of a Goal-Supporting mHealth App in Primary Care Among Patients With Multiple Chronic Conditions: Qualitative Descriptive Study
Source: JMIR Hum Factors. 2022 Nov 30;9(4):e37684. doi: 10.2196/37684 (PMC9752464; doi:10.2196/37684)
Supplement: Multimedia Appendix 1 [file humanfactors_v9i4e37684_app1.docx]

**Appendix 1:**

**Supplementary text: A: Two staged data analysis methods:**

**Stage 1:** **Deductive data analysis framework:** Deductive thematic analysis was conducted which allowed for a comprehensive understanding of the participants’ engagement behavior with the ePRO app related to SCT domains. We followed the six-step analysis strategy to analyze the study transcripts. Phase 1 involved two analysts (FT and TA) becoming familiar with the data through reading and re-reading the transcripts. During this initial stage, both analysts wrote down their initial thoughts and summaries of each transcript. Phase 2 included immersive coding where analysts generated a list of codes for SCT domains. This method aligns with a deductive approach in which the research team used established theoretical constructs to guide coding. To resolve any coding-related disagreement, the team met with the senior author (CSG) to reach a consensus. Codes were then collapsed into themes based on SCT core constructs, in Phase 3. Phase 4 involved the review of all themes and allowed the research team to comprehensively discuss the appropriateness of themes using a specific example from the transcripts. Phase 5 involved naming and providing a description per theme that was iteratively reviewed by the research team to assess the clarity of names and descriptions. At this stage, we have collapsed three domains of SCT goal efficacy, behavior capability, and outcome expectancies into one because it was identified that patients’ confidence in their goal, technological skills were linked with the anticipated outcome of the ePRO app. The final phase yielded a report, which is the focus of the results and discussion section. To enhance the rigor of this analysis, the research team members met regularly to discuss codes and findings. Having two data analysts helped ensure the dependability of the findings. ^1^

**Stage 2: Re-storying:**

After conducting deductive coding and collating the codes into subsequent themes, the authors identified that a deeper understanding of themes and their relationships to each other could be generated through “re-storying” of participants’ accounts chronologically. “Re-storying” is referred to as the method of rewriting participants’ oral data temporally to draw a link between prior experience with subsequent experiences. ^2^ To represent the finding of the current research study, “restorying” was necessary to draw the link between emerged themes in the deductive stage and sequencing of events to present a tangible explanation of the themes.

One of the major criticisms of SCT is that it does not recognize the wider social structure that influences an individual’s usage behavior. ^3^ The analytic method of re-storying used in this study address this challenge by the stories of long-term and short-term users that highlight the social contexts influencing their usage behavior. For example, the storylines allowed us to identify and outline major actors in the process (peers, providers), set goals (diet, exercise), complications (technical challenges, subsequent willingness to troubleshoot or not), and results (discontinuation or continuation with the app). Drawing this usage pathway was helpful to understand the core role providers and environment play in patients’ interest in adopting and adhering to a mHealth app.

Drawing on narrative methods, we constructed a matrix of themes distinguished between long-term and short-term app users (**The data matrix table can be found below in this appendix, Table 1**). After examining both columns of long-term and short-term app users, two research team members created one storyline for each group that captured the experience of the overall group. Throughout the re-storying process, both researchers discussed the accuracy of the storyline. Member checking was conducted 50 with study participants to examine the accuracy of the two storylines and overall interpretation of the study findings.

Bibliography:

1. Lincoln YS, Guba EG. Establishing Trustworthiness. Naturalistic inquiry. In: *Naturalistic Inquiry*. ; 1985.

2. Ollerenshaw jo A, Creswell JW. Narrative research: A comparison of two restorying data analysis approaches. *Qual Inq*. 2002;8(3):329-347. doi:10.1177/10778004008003008

3. Beauchamp MR, Crawford KL, Jackson B. Social cognitive theory and physical activity: Mechanisms of behavior change, critique, and legacy. *Psychol Sport Exerc*. 2019;42:110-117. doi:10.1016/j.psychsport.2018.11.009

**Table 1: Example of attributes of long-term and short-term app users**

| **Theme** | **Example attributes of short-term app users** | **Example attributes of long-term app users** |
| --- | --- | --- |
| **Reciprocal Determinism** | - Patients had an overall positive relationship with their provider   **Example:** Patient thought it would be more helpful if their provider could communicate via ePRO based on their goal progress (Patient #22)   - A communication feedback loop with their providers would be helpful for patients   **Example:** Patient thought their data is going into a *void* and no one is looking at it (Patient #15)   - Bad weather or personal injury often hindered patients’ progress towards the set goals, which negated patients’ need to use the app. (Patient #10) | - Patients had a trustworthy relationship with their providers - Patients have a close rapport with their provider which goes beyond the provider-patient relationship   **Example:** patient and their provider go to the same church where they volunteer together for social events (Patient #21) |
| **Goal Efficacy, Behavior capability and Outcome Expectancies** | - Some patients did not feel the app was ready to be implemented in a real-life setting and so they did not expect the app to be useful in their goal-tracking.   **Example:** “I never set goals, I think I am doing fine always. But my physician suggested I use this thing (ePRO) but I didn’t see much use. I do not need another thing, I barely use my own phone” (Patient #11) | - Patients relied on the enabling self-traits that will help them to achieve a set goal   **Example** : “I guess I am more of a self-motivated person rather than having to have something external to motivate me. But still very good app have to see how I am doing every week." (Patient # 20) |
| **Usage Reinforcement** | • Discouragement from the providers to use ePRO because health priority has changed  • Having a legacy device when ePRO may feel redundant  **Example:** “I already used my notebooks to write my health stuffs, so my dietician said forget the app, keep doing what you are doing.” (patient #13) | - Internal satisfaction of reaching goals   **Example***: "Well, to be honest, the only thing it did was every – I do it (check off the list), used to do it every Monday morning, and it focused me on not smoking. That was the motivation every Monday morning, you know"* (Patient #19) |
